# High Efficiency Inorganic/Inorganic Amorphous Silicon/Heterojunction Silicon Tandem Solar Cells

**DOI:** 10.1038/s41598-018-33734-y

**Published:** 2018-10-18

**Authors:** Jinjoo Park, Vinh Ai Dao, Sangho Kim, Duy Phong Pham, Sunbo Kim, Anh Huy Tuan Le, Junyoung Kang, Junsin Yi

**Affiliations:** 1 College of Information and Communication Engineering, Sungkyunkwan University (SKKU), Suwon, Kyunggi 440-746 Korea; 2 Institute of Fundamental and Applied Sciences, Duy Tan University, Ho Chi Minh City, 700000 Vietnam; 3 Ho Chi Minh City University of Technology and Education, 01 Vo Van Ngan, Thu Duc District, Ho Chi Minh City, 700000 Vietnam; 4 Department of Energy Science, Sungkyunkwan University (SKKU), Suwon, Kyunggi 440-746 Korea

## Abstract

We investigated high-efficiency two-terminal tandem photovoltaic (PV) devices consisting of a p/i/n thin film silicon top sub-cell (p/i/n-TFS) and a heterojunction with an intrinsic thin-layer (HIT) bottom sub-cell. We used computer simulations and experimentation. The short-circuit current density (J_sc_) of the top sub-cell limits the J_sc_ of the p/i/n-TFS/HIT tandem PV device. In order to improve the J_sc_ of the top sub-cell, we used a buffer-layer at the p/i and i/n interface and a graded forward-profile (f-p) band gap hydrogenated amorphous silicon germanium active layer, namely *i*-layer, in the top sub-cell. These two approaches showed a remarkable raise of the top sub-cell’s J_sc_, leading to the increase of the J_sc_ of the PV tandem device. Furthermore, in order to minimize the optical loss, we employed a double-layer anti-reflective coating (DL-ARC) with a magnesium fluoride/indium tin oxide double layer on the front surface. The reduction in broadband reflection on the front surface (with the DL-ARC) and the enhanced optical absorption in the long wavelength region (with the graded f-p band gap) resulted in the high J_sc_, which helped achieve the efficiency up to 16.04% for inorganic-inorganic c-Si-based tandem PV devices.

## Introduction

Significant energy losses due to the thermalization loss of photo-excited carriers having an energy greater than the bandgap, and the transmission loss of photons having energy less than the band gap, are inevitable in single-junction photovoltaic (PV) devices^1,2^. To overcome these drawbacks, researchers have used tandem PV device structures, which stacked multiple junctions having different band gaps in series. Among the tandem PV devices proposed, c-Si-based tandem PV devices have attracted considerable research interests due to their low cost, top sub-cells with tunable optoelectronic properties, and a possible efficiency of as high as 33% in a bifacial configuration^2,3^. To achieve high performance in these tandem solar cells, providing an excellent tunneling-recombination-junction is one of the most important design criteria^4–6^. Another key criterion involves the appropriate choice of top and bottom sub-cells, so that current matching among these sub-cells exists with the same short-circuit current density (J_sc_)^5^.

**Figure 1:**
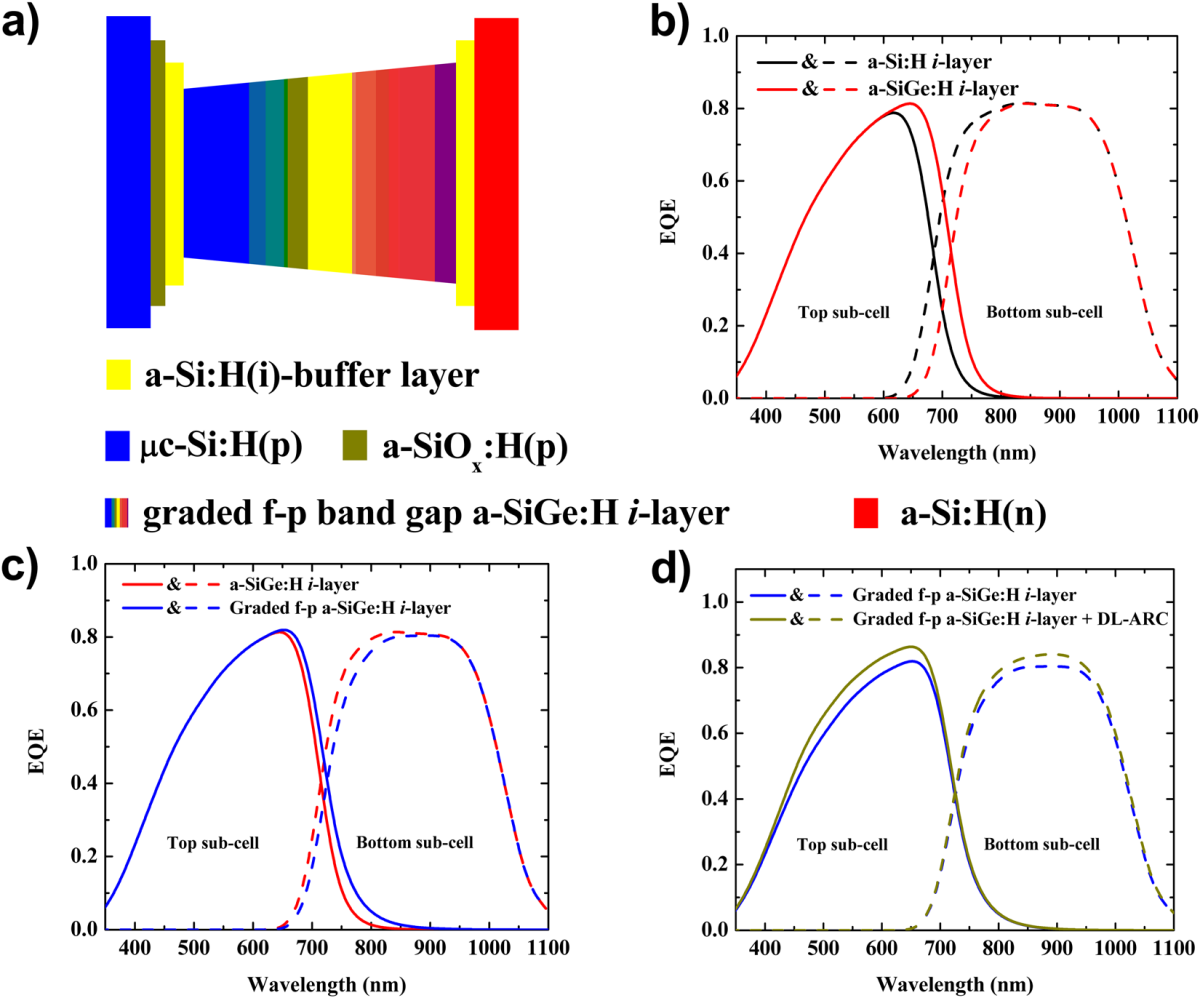
(**a**) Schematic band diagram of the top sub-cells with a graded forward band gap -profile (f-p) of the a-SiGe:H active layer with a buffer layer at the interfaces. (**b**–**d**) Simulated EQE spectra of the top (solid line) and bottom (dash line) sub-cell in the tandem cell with different materials and a profile band gap of the top sub-cell active layer having a thickness of 600 nm. Here, the black, red, blue, and dark-yellow lines indicate the EQE of each sub-cell of the tandem cell fabricated using various active *i*-layers for the top sub-cell as follows: a-Si:H, a-SiGe:H, graded f-p a-SiGe:H, and graded f-p a-SiGe:H active *i*-layer top sub-cell + DL-ARC, respectively.

In a tandem c-Si-based configuration, designers use c-Si having a band gap of around 1.1 eV as the bottom sub-cell, while the top sub-cell should have a band gap in the range of 1.4–1.8 eV^7,8^. Both organic-inorganic (perovskite/c-Si multi-junction) and inorganic-inorganic [p/i/n thin film silicon (p/i/n-TFS)/c-Si multi-junction] solar cell configurations have been investigated widely to achieve these band gap ranges^3,5,7–11^. In these devices, the tunneling-recombination junction plays a significant role in enhancing power conversion efficiency (PCE)^9^. Although the organic-inorganic tandem solar cells show higher initial device efficiency, the inorganic-inorganic type tandem devices are expected to show better stabilized performance in the long run. A relatively lower PCE in the inorganic-inorganic configuration might be attributed to the current mismatch between the two sub-cells, insufficient tunneling and recombination junction contact or both. Researchers expect that the device performance could be further improved by using improved *i*-layer of the top sub-cell of this tandem solar cell. In the inorganic-inorganic configuration, the p/i/n-TFS top sub-cell is a current limiting sub-cell; it means that the J_sc_ of the tandem device is approximated to the J_sc_ of the top sub-cell, as it produces less current. Therefore, improving the current density of the top sub-cell can lead to the higher tandem device’s J_sc_, which can be achieved by choosing appropriated light-trapping configuration materials, and profiling of the band gap of the *i*-layer of the top sub-cell. It is well-known that hydrogenated amorphous silicon germanium (a-SiGe:H) and hydrogenated nanocrystalline silicon (nc-Si:H) possess a higher absorption coefficient in the long wavelength region than hydrogenated amorphous silicon (a-Si:H)^12–17^. However, the narrow optical band gaps of an a-SiGe:H and nc-Si:H could cause band gap discontinuity at the p/i and i/n interfaces, leading to a detrimental open-circuit voltage (V_oc_) and fill factor (FF) due to the high defect density at these interfaces^18,19^. Thus, we propose the possibility of sustaining high V_oc_, FF, and J_sc_ simultaneously by using the band gap profiling of the highly absorbing a-SiGe:H alloy, along with buffer layers at the p/i and i/n interfaces, as shown in Fig. 1(a).

In this study, we report a tandem solar cell having a buffer-layer at the (p/i and i/n) interfaces and band gap profiling of the active *i*-layer in the top sub-cell. Magnesium fluoride (MgF_2_)/indium tin oxide (ITO) was employed as a double-layer anti-reflective coating (DL-ARC) for the light-trapping configuration. In the first part of this study, we performed optical simulations in combination with experimental data related to the optical properties of each layer. We utilized the simulated results to help design an optimal tandem device structure. In the latter part, we fabricated tandem solar cells based on the simulated results. We investigated the effects of the materials, the band gap profiles (interface and active *i*-layer), and the DL-ARC on the J_sc_. The PCE of the optimized p/i/n-TFS/heterojunction with an intrinsic thin-layer (HIT) tandem solar cell showed the J_sc_ of up to 15.19 mA/cm^2^ and an efficiency of 16.04%, representing the highest PCE to date of the tandem solar cell based on an inorganic-inorganic c-Si-based tandem solar cell.

## Results

**Figure 2:**
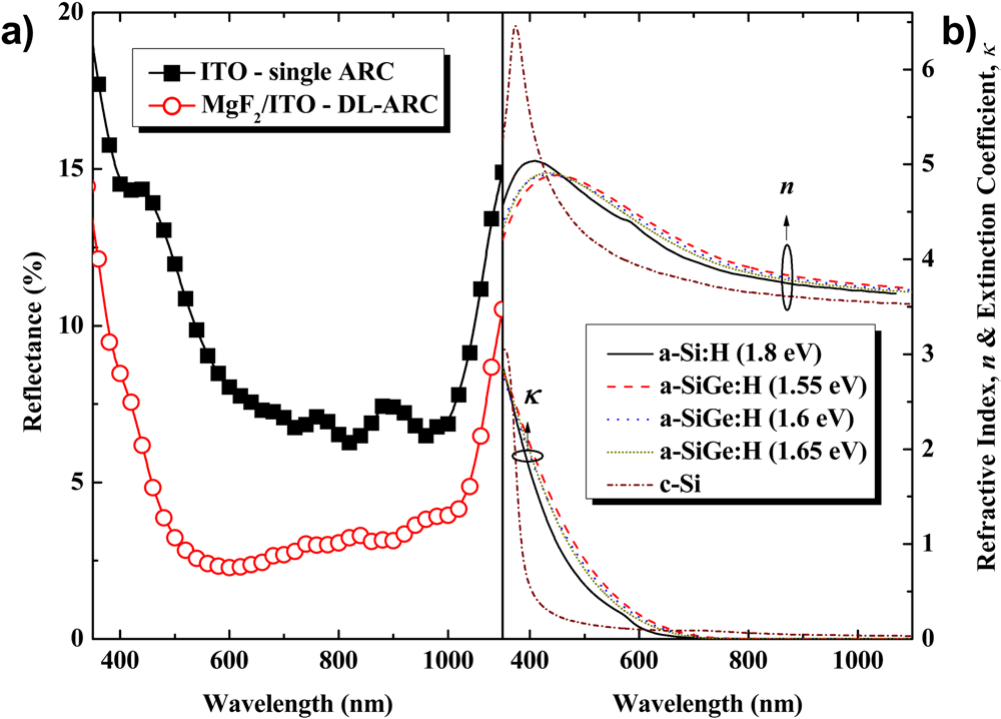
(**a**) Surface reflectance of the tandem device, with ITO as a single anti-reflective coating (ARC) and MgF_2_/ITO as a double-layer anti-reflective coating (DL-ARC). (**b**) Optical constants, ***n*** and ***κ***, of the a-Si:H(i) [band gap (E_g_ = 1.8 eV)] (solid black line); a-SiGe:H(i) [E_g_ = 1.55 eV] (dashed red line); a-SiGe:H(i) [E_g_ = 1.60 eV] (dotted blue line); a-SiGe:H(i) [E_g_ = 1.65 eV] (short dot dark-yellow line) and c-Si (short dashed-dotted dark red line).

**Figure 3:**
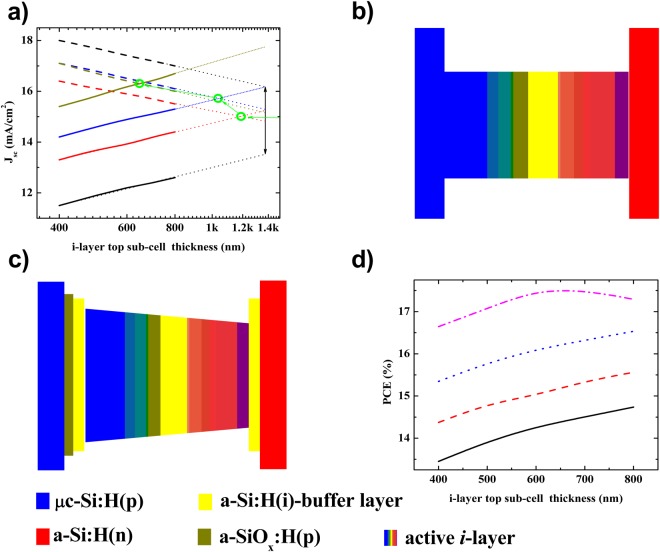
(**a**) Plot of photo-generated current-density of the top (solid line) and bottom (dashed line) sub-cells in the tandem PV device, as a function of the active *i*-layer thickness of the top sub-cell; the green open-circle points represent the J_sc_ matching condition. Here, the black, red, blue, and dark-yellow lines indicate the J_sc_ of each sub-cell of the tandem cell fabricated using various active *i*-layer of the top sub-cell as follows: a-Si:H(i), a-SiGe:H, graded f-p a-SiGe:H, and graded f-p a-SiGe:H active *i*-layer top sub-cell + DL-ARC, respectively. (**b**) Schematic band diagram of the top sub-cells with a-Si:H or a-SiGe:H active *i*-layer constant-profile band gap. (**c**) Schematic band diagram of the top sub-cells with a-SiGe:H active *i*-layer graded reverse-profile (r-p) band gap with a buffer layer at the interfaces. (**d**) Predicted PCE of the tandem PV devices as a function of the active *i*-layer thickness of the top sub-cell; here, the solid black, dashed red, dotted blue, and dashed-dotted magenta colored lines indicate the PCE of the tandem PV device fabricated using a-Si:H, a-SiGe:H, graded f-p a-SiGe:H, and graded f-p a-SiGe:H active *i*-layer for top sub-cell + DL-ARC, respectively.

Figure 1(b–d) depict the calculated external quantum efficiency (EQE) of each sub-cell in the tandem solar cell; here, the thickness of the active *i*-layer of the top sub-cell was fixed at 600 nm. In this study, we calculated the EQE of each sub-cell in the p/i/n-TFS/HIT tandem solar cells using the Schade and Smith method^20^, based on the optical properties of all layer components and device geometries. The Schade and Smith method can be expressed as:

1 $$EQE(\lambda )=[1-{R}_{F}(\lambda )]\,[1-{A}_{ARC}(\lambda )]\,[1-{A}_{p}(\lambda )]\,[1-{A}_{n}(\lambda )]\,[{A}_{i}(\lambda )]$$

where *R*_*F*_ is the front reflectance (measured by a UV-Vis spectrophotometer) (Fig. 2(a)) and *A* is the absorbance. The subscripts *ARC*, *p*, *n*, and *i* (in the case of HIT-type bottom sub-cell, we used the n-type c-Si instead of the *i*-layer) are the relevant layer components in the tandem cell. For *A*(*λ*) = 1 − exp[−*α*(*λ*)*d*], *α*(*λ*) and *d* are the absorption coefficients and the film thickness of each layer component, respectively. To calculate the optical absorption of each layer, we measured the refractive index (***n***) and extinction coefficient (***κ***) for the respective layers using ellipsometry, as depicted in Fig. 2(b). It is worth noting that we did not consider the back-reflection on the back surface of the device in this calculation, and assuming that all of the rays entering the device contributed entirely for the quantum efficiency of the tandem cell. Thus, the J_sc_ of each sub-cell was calculated according to:

2 $${J}_{sc}=q\,\int \,EQE(\lambda ).I(\lambda ).d\lambda $$

where *q* is the elementary charge and *I*(*λ*) is the spectra irradiance of incident light. From the EQE spectra of each sub-cell respective to an active *i*-layer thickness presented on Fig. 1(b–d), we performed the calculated the J_sc_ with regard to each active *i*-layer thickness. Figure 3(a) shows the calculated J_sc_ of the top-cell (solid line) and the bottom-cell (dash line) as a function of the active *i*-layer top sub-cell thickness. Here, the black-, red-, blue-, and dark yellow-color indicate the J_sc_ sub-cells of the tandem cell fabricated using a standard constant-profile band gap a-Si:H *i*-layer top sub-cell (Fig. 3(b)), a constant-profile band gap a-SiGe:H *i*-layer top sub-cell (Fig. 3(b)), a graded forward-profile (f–p) band gap a-SiGe:H *i*-layer top sub-cell (Fig. 1(a)), and a graded f-p band gap a-SiGe:H *i*-layer top sub-cell + DL-ARC, respectively. Henceforth, we use a-Si:H *i*-layer and a-SiGe:H *i*-layer to indicate a constant-profile band gap of a-Si:H *i*-layer and a-SiGe:H *i*-layer, respectively. It is worth noting that without MgF_2_/ITO as a DL-ARC, the cells were ordinary tandem solar cells with ITO as a single layer ARC. The green open-circle points represent the intersection of J_sc_ between the top and bottom sub-cells, with the intersections exhibiting the same J_sc_ between the top and bottom sub-cells. Comparing to the standard a-Si:H(i) active-layer, the high absorption a-SiGe:H(i) layer showed slightly higher tandem cell’s J_sc_, while the *i*-layer thickness decreased considerably. Thanks to this low thickness, the a-Si:Ge:H(i) layer took many merits of saving deposition material and lowering light-induced degradation effect. However, the high absorption of the a-SiGe:H *i*-layer may have been detrimental to the photon yield in the bottom sub-cell; hence, the J_sc_ of the bottom sub-cell significantly decreased [red dashed line in (Fig. 3(a)), which then potentially limited the J_sc_ of the tandem device. To overcome this weakness, we used the buffer layer at the interfaces (p/i and i/n) and the graded f-p band gap active *i*-layer for the top sub-cell (Fig. 1(a)). The J_sc_ increased by about 4% compared to the device without buffer layer at the interfaces (p/i and i/n) and the graded f-p band gap *i*-layer. Finally, by adding the DL-ARC to the top of the tandem cell (whereby the top sub-cell comprised of the buffer layer at the interfaces (p/i and i/n) and the graded f-p band gap active *i*-layer), we could obtain a calculated value for tandem cell J_sc_ up to 16.2 (mA/cm^2^).

Since the top and bottom sub-cells were electrically in series, the J_sc_ of the whole tandem cell could be identified by the lower J_sc_ (top/bottom) value^21^. The V_oc_ of the tandem cell would be equal to the sum of the V_ocs_ of the sub-cells, while we expected the FF of the tandem cell to be the mean value of the top and bottom sub-cells^2^. The predicted PCE for the p/i/n-TFS/HIT tandem solar cells is shown in Fig. 3(d), indicating that the highest PCE value could be obtained when the current matching condition was fulfilled. The device fabricated with the buffer layers at the interfaces and the graded f-p band gap a-SiGe:H + DL-ARC showed the highest performance among the calculated tandem cells, with the thinnest top sub-cell thickness of around 600 nm was used. We then used this value as a reference thickness for device fabrication.

**Figure 4:**
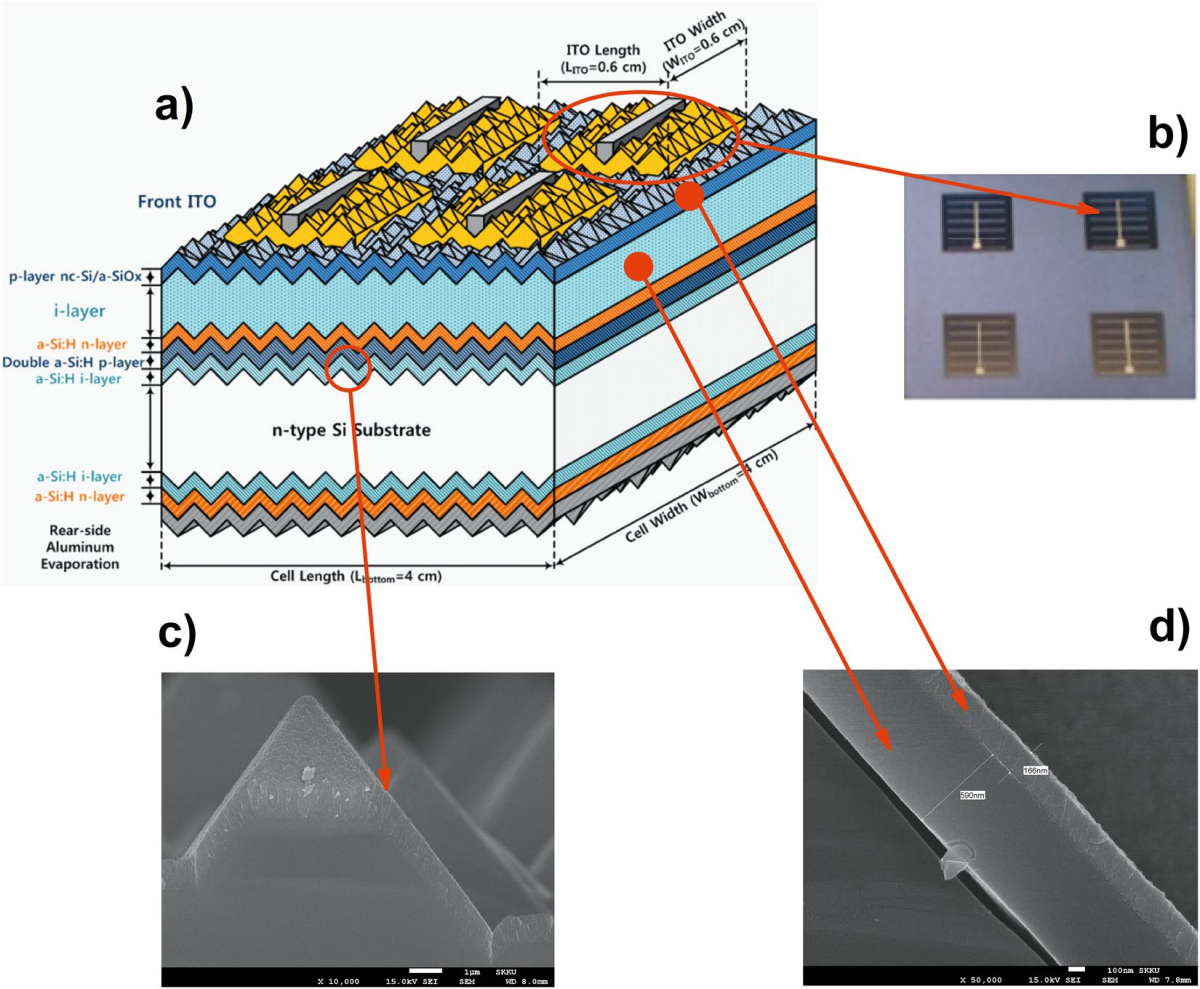
(**a**) Schematic diagram showing the configuration of the tandem solar cell consists of a p/i/n-TFS top sub-cell and a HIT-type bottom sub-cell. (**b**) Top-view of tandem solar cell graphed by an optical microscope. (**c**) Cross-section SEM image of the pyramidal surface texture of the anisotropic wet-chemical etching of the n-type c-Si substrate. (**d**) Cross-section SEM image of the p/i/n-TFS top sub-cell on a rough texture similar to that of the bottom cell.

To confirm the calculated results, we fabricated a tandem solar cell consisting of a p/i/n-TFS top sub-cell and a HIT-type bottom sub-cell (Fig. 4(a,b)). We realized different active *i*-layers for the top sub-cell (e.g. the standard a-Si:H, the a-SiGe:H, and the graded f-p bandgap a-SiGe:H). We retained the thickness of the active *i*-layer of the top sub-cell fixed at 600 nm, as mentioned previously. Finally, to attain a higher J_sc_ for the tandem cell, and thus a higher PCE, we employed the DL-ARC on the tandem cell having a graded f-p band gap a-SiGe:H *i*-layer top sub-cell. Figure 4(c) depicts a cross-sectional scanning electron microscopic (SEM) image of the pyramidal surface texture of the anisotropic wet-chemical etching of the n-type c-Si substrates, while Fig. 4(d) depicts a cross-sectional SEM image of the p/i/n-TFS top sub-cell on a rough texture similar to that of the bottom cell.

**Figure 5:**
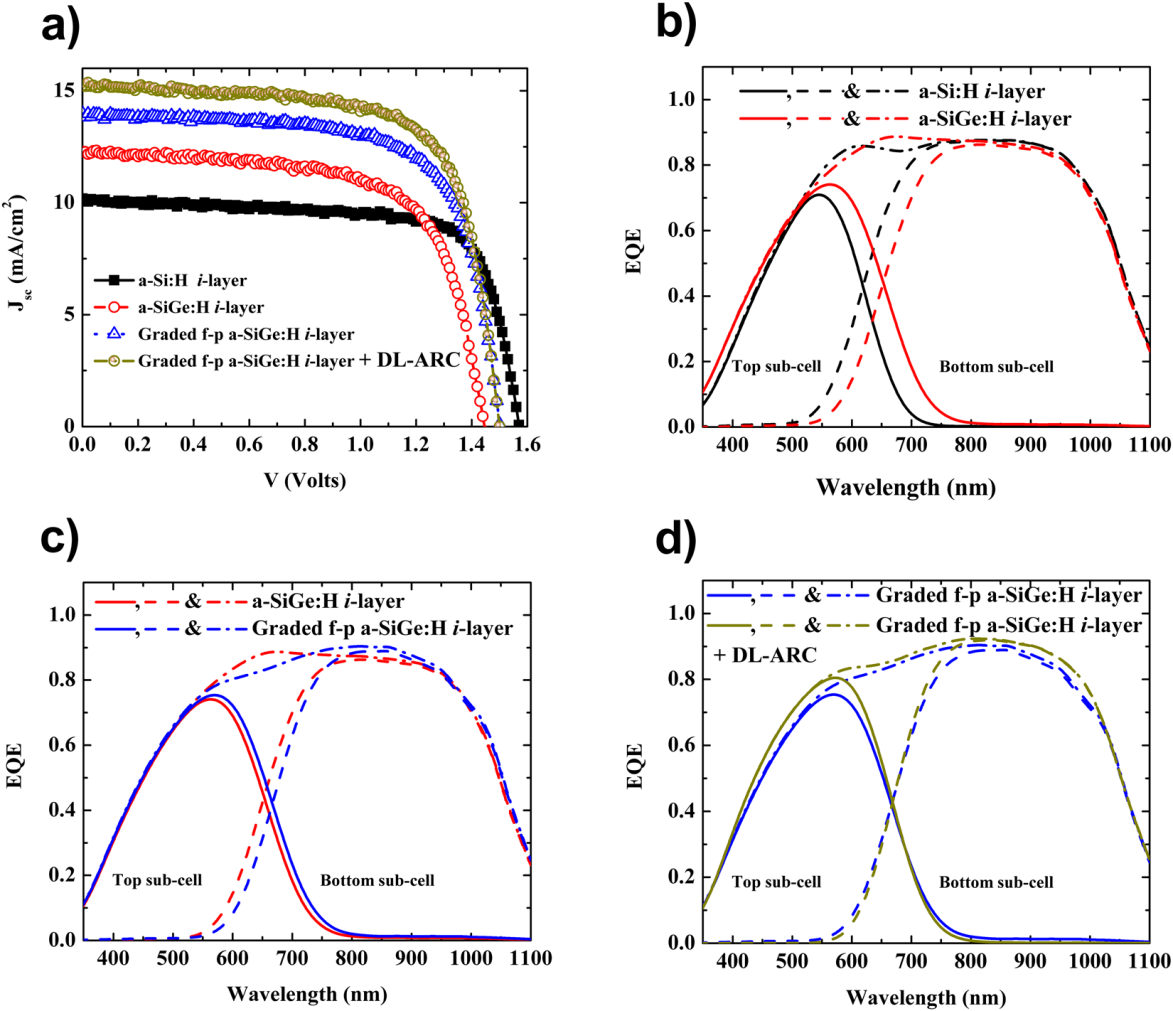
(**a**) J-V characteristics of the tandem solar cells for different active *i*-layer top sub-cells and for graded f-p band gap a-SiGe:H active *i*-layer top sub-cells + DL-ARC. (**b**–**d**) Measured EQE spectra of the top (solid line) and bottom (dashed line) sub-cell, and the tandem cell (dashed-dotted line) with different materials and band gap profiled active *i*-layer having a top sub-cell thickness of 600 nm. Here, the back, red, blue, and dark-yellow lines indicate the measured EQE of each sub-cell and the tandem cell with respect to the tandem cell fabricated using the a-Si:H active *i*-layer, a-SiGe:H active *i*-layer, graded f-p a-SiGe:H active *i*-layer, and graded f-p a-SiGe:H active *i*-layer + DL-ARC, respectively, for the top sub-cell.

The individual measurements of the top and bottom sub-cell are shown in Table 1. In this Table, the p/i/n-TFS top sub-cells show high V_oc_ and low J_sc_ compared to those of a HIT-type bottom sub-cell. These properties are important for eliminating or reducing thermalization losses in the tandem solar cell. In Table 1, we also summarize the experimental performances (e.g., J_sc_, V_oc_, FF, and η) of the tandem solar cells having the a-SiGe:H *i*-layer top sub-cell, the graded f-p bandgap a-SiGe:H *i*-layer top sub-cell, and the graded f-p band gap a-SiGe:H *i*-layer top sub-cell employing MgF_2_/ITO as a DL-ARC. The corresponding J-V characteristics are shown in Fig. 5(a). For comparison, Table 1 and Fig. 5(a) also present the parameters for a tandem solar cell with the standard a-Si:H *i*-layer top sub-cell, showing that the performance of the tandem solar cells changed considerably with the variation in the *i*-layer of the top sub-cell. The tandem solar cell with the standard a-Si:H *i*-layer top sub-cell showed the V_oc_ of 1.56 V, the J_sc_ of 9.83 mA/cm^2^, the FF of 76.02%, and the PCE of 11.65%. These results are obviously better than those obtained in investigations of similar tandem configurations reported in the literature^5,9,22^, yet remain lower than the PCE of organic-inorganic silicon-based tandem cells^3,8,10,11^. The tandem solar cell fabricated with the a-SiGe:H *i*-layer top sub-cell showed a significant increase in J_sc_ from 9.83 to 12.26 mA/cm^2^, while obtaining a remarkable decrease in V_oc_ and FF from 1.56 (V) and 76.02% to 1.44 (V) and 67.01%, respectively, thereby leading to a slight increase of PCE from 11.65% to 11.84%. Thus, the use of an active a-SiGe:H *i*-layer, which is not only a high absorption in the red region but also a narrow band gap material, yields high J_sc_, yet was detrimental to the V_oc_ and FF; hence, the overall device efficiency enhancement was insignificant. To minimize the electrical and optical losses simultaneously, we employed buffer layers at the interfaces (p/i and i/n) and the graded f-p band gap of the a-SiGe:H *i*-layer for the top sub-cell, as presented in Fig. 1(a). With this approach, the V_oc_, J_sc_, and FF increased from 1.44 (V), 12.26 (mA/cm^2^), and 67.01 (%) to 1.50 (V), 13.82 (mA/cm^2^), and 70.05 (%), respectively. Hence, we achieved a cell efficiency of 14.52%. For further improvement in light trapping, we employed MgF_2_/ITO on the top of the device to serve as a DL-AR coating. This helped improve the J_sc_ significantly from 13.82 (mA/cm^2^) to 15.19 (mA/cm^2^) and boost the PCE of the tandem solar cell to 16.04%. Here, the V_oc_ and FF values did not change remarkably compared to those without the MgF_2_ layer. This PCE value represents the highest to date for a tandem solar cell base among inorganic-inorganic Si-based configurations.

**Table 1:** Performances of the tandem solar cell having the J-V characteristics in Fig. 5(a).

Device	V_oc_ [V]	J_sc_ [mA/cm^2^]	FF (%)	PCE [%]
HIT	0.71	38.77	75.42	20.78
p/a-Si:H(i)/n-TFS	0.94	14	72	9.47
p/a-SiGe:H(i)/n-TFS	0.84	18.4	65.0	10.05
p/graded f-p band-gap a-SiGe:H(i)/n-TFS	0.83	18.8	68.0	10.61
p/a-Si:H(i)/n-TFS/HIT	1.56	9.83	76.02	11.65
p/a-SiGe:H(i)/n-TFS/HIT	1.44	12.26	67.01	11.84
p/graded f-p band-gap a-SiGe:H(i)/n-TFS/HIT	1.50	13.82	70.05	14.52
p/graded f-p band-gap a-SiGe:H(i)/n-TFS/HIT + DL-ARC	1.50	15.19	70.31	16.04

In addition, Fig. 5(b–d) depict the measured EQE of each sub-cell in the tandem solar cells; the results are in good agreement with the simulated EQE values (Fig. 1(b–d)). Here, the experimental EQE results of the bottom sub-cells were the higher than those of the top sub-cells; whereas the simulated EQE results of the two sub-cells were relatively similar. This difference may be attributed to the back-reflection of the bottom sub-cell that was not considered in the simulation. The EQE of the top sub-cell using buffer layers at the interfaces (p/i and i/n) and the graded f-p band gap a-SiGe:H *i*-layer improved significantly in the red-wavelength region compared to that of the top sub-cell using the standard a-Si:H and/or a a-SiGe:H *i*-layer. On the other hand, the EQE of the bottom sub-cell decreased in this given wavelength when we replaced the standard a-Si:H *i*-layer top sub-cell with a-SiGe:H or the graded f-p band gap a-SiGe:H *i*-layer. Figure 5(b–d) also showed the measured total EQE of the tandem cells. The behavior of the total EQE of the tandem-cell showed an improvement in the wavelength range of 650 to 800 nm (when a-SiGe:H was used instead of a-Si:H) and 750 to 1000 nm (when the graded f-p band gap a-SiGe:H was used instead of the constant band gap a-SiGe:H). However, there was a reduction in the total EQE of the tandem-cell in the wavelength range of from 500 to 650 nm (when a-SiGe:H was used instead of a-Si:H), and 550 to 750 nm (when graded f-p band gap a-SiGe:H was used instead of a-SiGe:H). Hence, it appeared that the EQE of the tandem-cell did not change considerably as varied the active-layer materials and configuration. Interestingly, using DL-ARC, the EQE of all sub-cells as well as the tandem-cell enhanced remarkably in all wavelength ranges of interest.

## Discussions

**Figure 6:**
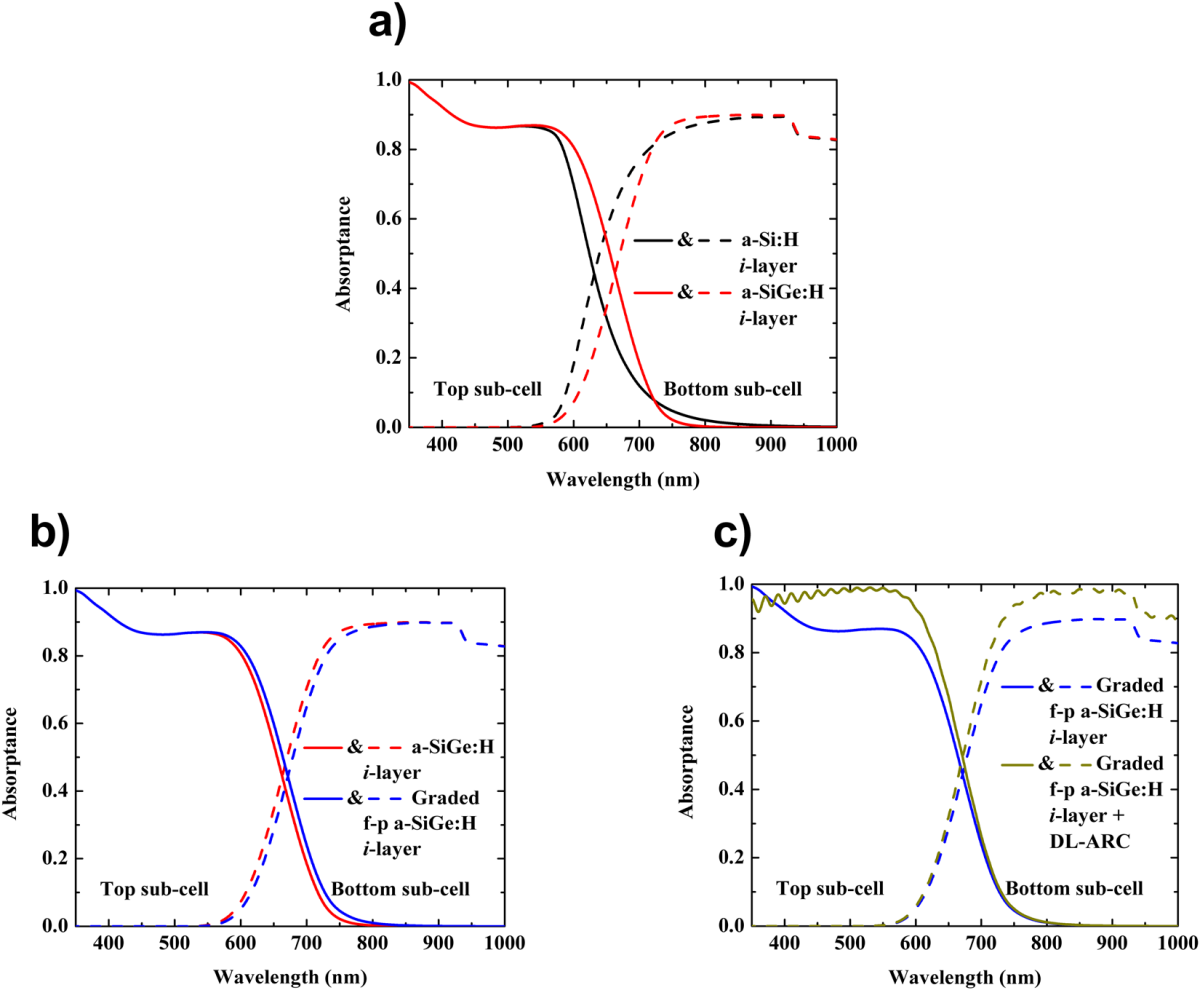
Absorptance plots of the top (solid line) and bottom (dashed line) sub-cell in the tandem solar cells having different active *i*-layers of the top sub-cells. Here, the black, red, blue, and dark-yellow lines indicate the absorptance plots of each sub-cell in the tandem cell fabricated using a-Si:H, a-SiGe:H, graded f-p profile band gap a-SiGe:H, and graded f-p band gap a-SiGe:H + DL-ARC, respectively, for the active *i*-layer top sub-cell.

As presented in Fig. 6(a), the standard a-Si:H *i*-layer with 1.8 (eV) band gap as the active-layer in the top sub-cell suffered from the absorption loss at long wavelengths (600 nm < λ < 800 nm), while the absorption behavior in the HIT-type bottom sub-cell revealed the opposite trend to that of the top sub-cell in this given wavelength range. The calculated EQE in Fig. 1(b) and the calculated J_sc_ in Fig. 3(a) demonstrated the low absorption of the top sub-cell and the high absorption of the bottom sub-cell. Due to the J_sc_ mismatch, the J_sc_ of the top sub-cell limited the overall J_sc_ of the tandem cell. Therefore, we employed a high absorption a-SiGe:H layer with band gap of 1.6 (eV) as an active layer for the top sub-cell; this reduced the discrepancy in J_sc_ between the two sub-cells and exhibited an improvement in the J_sc_ of the tandem cell. Nevertheless, the thickness of the top sub-cell remained relatively high (~1200 nm), leading to poor photon harnessing in the wavelength range of 550–800 nm by the HIT-type bottom sub-cell, which then lowered the J_sc_ of the tandem device (Fig. 3(a)).

In order to enhance the light absorption process in the top sub-cell without hindering the light absorption in the bottom sub-cell, we considered the multi-profiling band gap of the a-SiGe:H *i*-layer of the top sub-cell. We first performed simulations for three-types of the graded band gap profile of the *i*-layer for only single p/i/n-TFS, including a-SiGe:H (Fig. 3(b)), the graded f-p band gap a-SiGe:H (Fig. 1(a)), and a graded reverse-profile (r-p) band gap a-SiGe:H (Fig. 3(c)). For these simulations, we utilized ASA software^23^. We set the simulation parameters of all layers in the single p/i/n-TFS to be the same as in the top sub-cell of the tandem. The simulated results are shown in Table 2. The cell with the graded f-p band gap exhibited the highest J_sc_, yet the lowest V_oc_. In contrast, the cell with the graded r-p band gap showed the highest V_oc_, but the lowest J_sc_. These simulation results appear to be consistent with the experimental results reported by Cao *et al*.^24^ and Guha *et al*.^25^. The improvement of J_sc_ for the graded f-p bandgaps *i*-layer may be attributed to the enhancement of light absorption in the infrared region and to an additional built-in field due to the band gap profile^24^. Hence, to further enhance top sub-cell absorption, we considered a multi-profiled band gap with an f-p configuration of the a-SiGe:H *i*-layer for the top sub-cell in order to calculate the absorptance in the tandem cells. The absorptance spectra for top (solid lines) and bottom (dash lines) sub-cells are plotted, with the red curve is for the a-SiGe:H *i*-layer top-cell and the blue one is for the graded f-p band gap a-SiGe:H *i*-layer top-cell (Fig. 6(b)). The graded f-p band gap a-SiGe:H *i*-layer helped boost the absorptance spectra for the top sub-cell across the entire wavelength range of interest; meanwhile, the absorptance spectra for the bottom sub-cell showed the contrary trend. The calculated EQE (Fig. 1(c)) and calculated J_sc_ of the tandem cell (Fig. 3(a)) demonstrated the absorptance behaviors of the top and bottom sub-cells.

**Table 2:** Simulated device performances of the single p/i/n-TFS top sub-cell having different band gap profile active *i*-layers.

Devices	V_oc_ [V]	J_sc_ [mA/cm^2^]	FF (%)	PCE [%]
p/a-SiGe:H(i)/n-TFS	930	15.3	69.97	9.94
p/graded f-p band-gap a-SiGe:H(i)/n-TFS	897	17.0	64.38	9.81
p/graded r-p band-gap a-SiGe:H(i)/n-TFS	965	13.1	69.43	8.75

**Figure 7:**
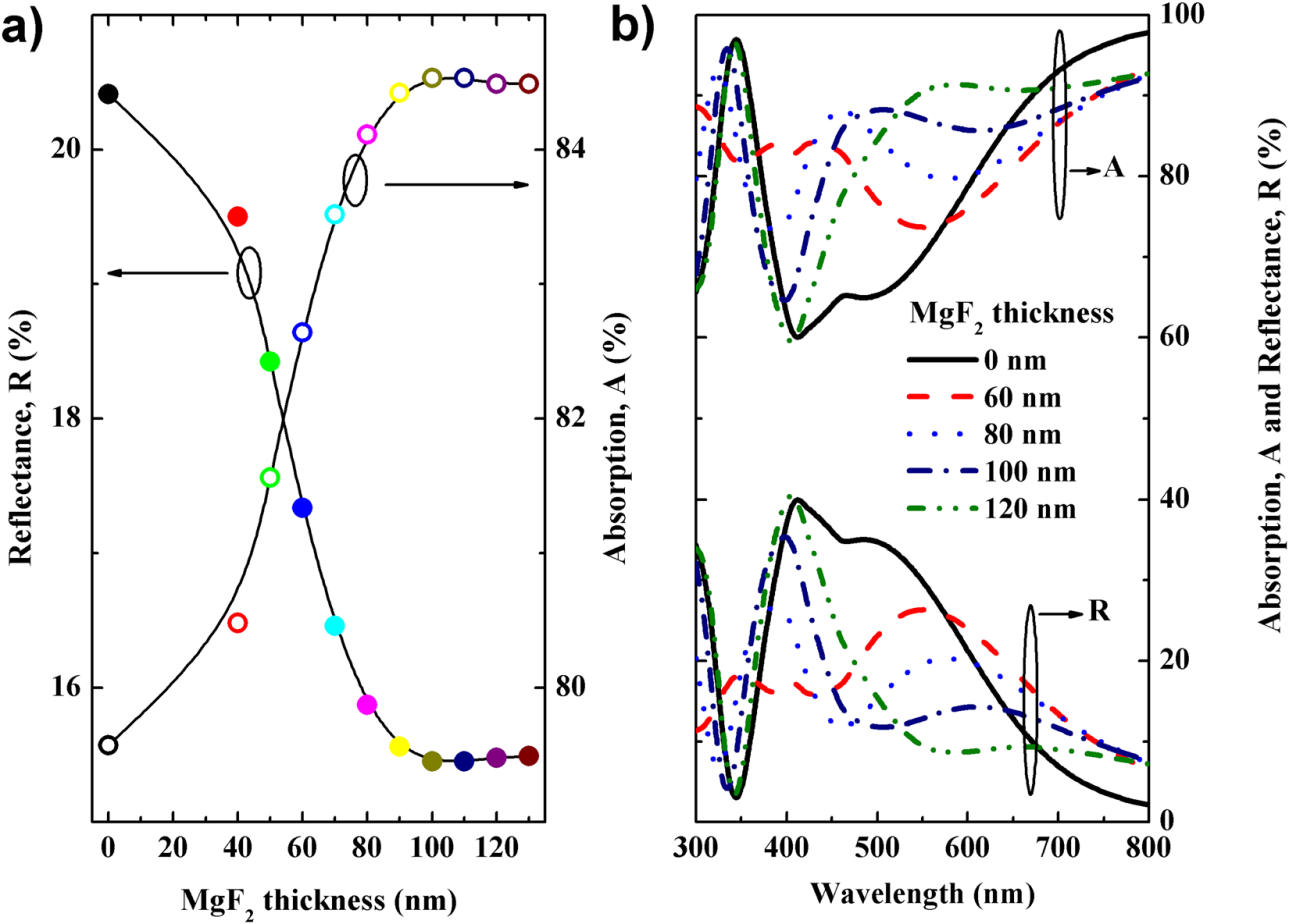
(**a**) Average reflectance and absorption of the MgF_2_/ITO DL-ARC as a function of the MgF_2_ layer thickness. (**b**) Reflectance and absorption of the MgF_2_/ITO DL-ARC as a function of the wavelength for varying MgF_2_ thickness.

To further minimize the optical loss in the tandem PV devices, we finally employed MgF_2_ layer on the top of the ITO, namely DL-ARC. First, we employed Macleod software to determine an optimum thickness of the MgF_2_ layer in the DL-ARC. Here, we retained the thickness of the ITO at 160 nm (as used in the experimental structure), while varying the thickness of MgF_2_ in the range of 0 to 120 nm. Thus, from (Fig. 7(a,b)), a thickness of 105 ± 5 nm for MgF_2_ appeared sufficiently to obtain the lowest reflectance. Therefore, the dielectric layer of the 105 ± 5 nm-thick MgF_2_ was used in the tandem cells to calculate its absorption. The absorptance spectra for the tandem cell with the DL-ARC layer are shown in Fig. 6(c), indicating that the DL-ARC could increase the absorption spectra across the entire wavelength range of interest for both the top and bottom cells; as such, the J_sc_ of the top and bottom cells both increased, and the J_sc_ of the tandem cell was thus enhanced significantly (Fig. 3(a)).

From Table 1 and Fig. 5(a), the experimental J_sc_ value for all three devices (the a-SiGe:H *i*-layer, the graded f-p band gap a-SiGe:H *i*-layer, and the MgF_2_ layer on the top tandem cell having a graded f-p band gap a-SiGe:H *i*-layer for the top sub-cell) showed a similar trend to those of the simulated calculations. We attributed this tendency of the J_sc_ of the tandem cell to the improvement of absorption spectra across the entire wavelength range of interest, which then improved the EQE, as discussed earlier. However, as seen in Table 1 and Fig. 5(a), well-known research has shown that the use of a narrow band gap a-SiGe:H *i*-layer for the top cell could cause band gap discontinuities (lattice mismatch between the n/i- and i/p-heterointerface) and high-defect densities at the p/i and i/n interfaces^19,25^, reducing the internal electric field and the carrier collection^26^, thereby reducing the V_oc_ and FF. In order to minimize the effective interface recombination losses, we employed buffer layers at the interface. A schematic of the buffer layers for the top sub-cell is shown in Fig. 1(a). The thickness and band gap of the a-Si:H(i) buffer layer were 10 nm and 1.80 eV at the n-layer side, respectively. The thickness and band gap of the a-Si:H(i) buffer layer at the p-layer side were 20 nm and 1.80 eV, respectively, and those of the a-SiO_x_:H(p) layer at the p-layer side were 10 nm and 2.1 eV respectively. Specifically, the thickness and band gap were 40 nm and 1.81 eV for the n-type a-Si:H,, and were 25 nm and 2.03 eV for the p-type μc-Si:H. Thus, by introducing a buffer layer at the interface, the tandem cell depicted significant improvement of V_oc_, J_sc_, and FF compared to that with no buffer layer. Consequently, the best cell configuration (corresponding to a graded f-p band gap a-SiGe:H *i*-layer with a profiled buffer layer inserted at the p/i and i/n interfaces, employing a DL-ARC) showed a high V_oc_ of 1.5 V, J_sc_ of 15.19 mA/cm^2^, and FF of 70.31%; the resulting PCE of 16.04% represents the highest obtained PCE to date for an inorganic-inorganic c-Si-based tandem solar cell.

From the device performances of each individual sub-cell and tandem cell shown in Table 1, it is observed that the J_sc_ of the tandem cell (15.19 mA/cm^2^) is lower than that in both the p/i/n-TFS (18.8 mA/cm^2^) and HIT (38.88 mA/cm^2^) cells. Thus, it is possible to obtain higher tandem cell efficiency without adding cost to the traditional tandem configuration, by carefully tuning the J_sc_ of the top and bottom sub-cells. It was suggested that a PCE of more than 36% can be obtained by thinning the top sub-cell so that it absorbs only 68% of incident photons, thus transmits 32% of the incident photons to the c-Si bottom sub-cell^27^. In this case, the optimal band gap of the top sub-cell is 1.46 eV. This band gap is very close to that of a-SiGe, as reported by S. Guha; a possible J_sc_ of as high as 22.4 mA/cm^2^ of the top sub-cell can thus be achieved^25^. Moreover, light absorption in the HIT bottom sub-cell can be improved by using a bifacial design^3^. This design can enhanced approximately 30% photon absorption into the HIT bottom sub-cell from the rear face. Consequently, the bifacial tandem cell enhances the J_sc_ of the HIT bottom cell. It is therefore possible to use a thicker p/i/n-TFS top sub cell to enhance J_sc_, and the J_sc_ of tandem cell consequently improves. Thus, the high efficiency of a p/i/n-TFS/HIT tandem solar cell of more than 28% (V_oc_ ≈ 1.6 V, J_sc_ ≈ 22 mA/cm^2^, FF ≈ 0.82) may be achievable, which is higher than the 24.7% individual HIT cell^28^.

**Figure 8:**
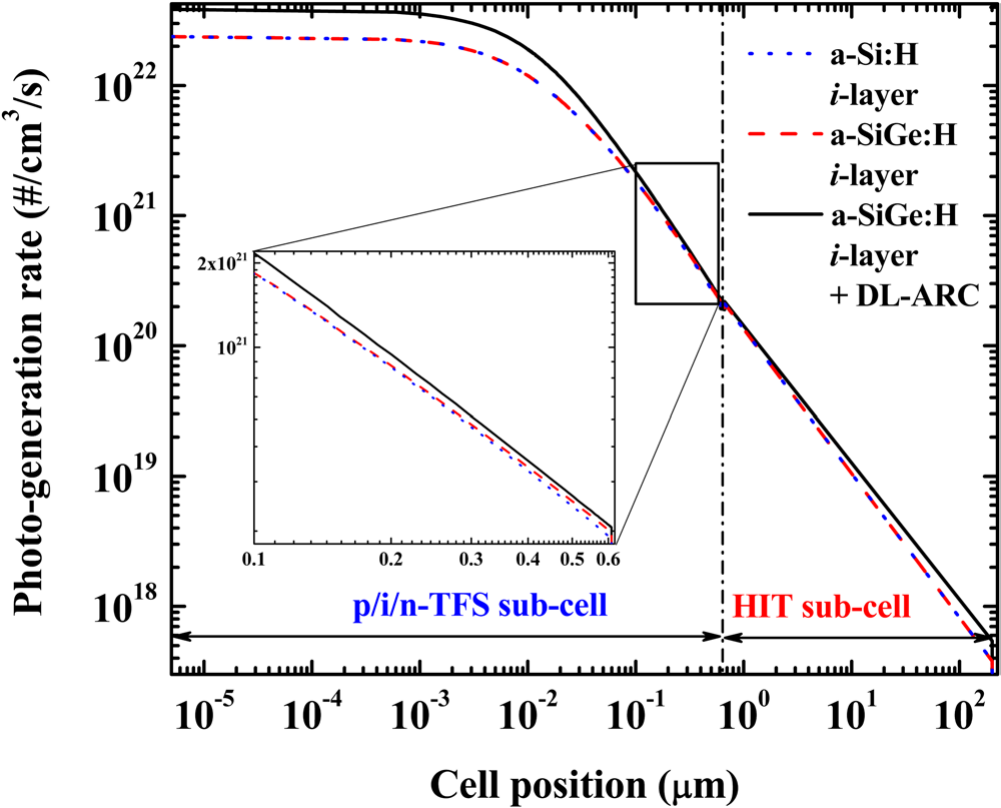
Plot of the photo-generation rate in a tandem solar cell with variation of the active *i*-layer top sub-cell, such as a-Si:H *i*-layer (dotted blue line), a-SiGe:H *i*-layer (dashed red line), and a-SiGe:H *i*-layer + DL-ARC (solid black line), as a function of the cell position.

We utilized the AFORS-HET software in order to understand the photo-generation rate phenomenon of the PV tandem devices^29^. Figure 8 depicts the simulated photo-generated rate distribution inside the PV tandem device for two different top sub-cell band gaps (a standard a-Si:H *i*-layer having a band gap of 1.8 eV and a a-SiGe:H *i*-layer having a band gap of 1.6 eV), and the PV tandem device having a top sub-cell using a-SiGe:H (band gap of 1.6 eV) + DL-ARC. Here, we were unable to simulate the tandem PV devices having a graded f-p band gap top sub-cell due to limitations in the current version of the AFORS-HET software. Compared to a standard a-Si:H *i*-layer top sub-cell, the tandem PV device with a narrow band gap of 1.6 eV showed a higher photo-generation rate in the top sub-cell (0.1 to 0.6 µm), as shown in the inset of Fig. 8. This higher photo-generation could be attributed to the higher extinction coefficient of the narrow band gap material. In addition, the photo-generation rate remarkably improved in both the top and bottom sub-cells employing a DL-ARC. This result explains the improvement of the both J_sc_ and PCE of the tandem PV device, as shown in Fig. 3(d) (simulation), Table 1, and Fig. 5(a) (experiment).

In summary, we carried out investigations of the influences of several key parameters on the performances of tandem solar cells (e.g. the materials of the active *i*-layer of the top sub-cell, the buffer layers at the p/i and i/n interfaces, the multi-profiling band gaps of the active *i*-layer of the top sub-cell, and the DL-ARCs). Due to its higher optical absorption, the constant band gap a-SiGe:H active *i*-layer for the top sub-cell (instead of a standard a-Si:H active *i*-layer) exhibited higher EQE in the long wavelength region. This substitution led to less discrepancy in the J_sc_ between the two sub-cells, thus increasing the short-circuit current density from 9.83 to 12.26 mA/cm^2^. However, this narrow constant-profiled band gap a-SiGe:H active *i*-layer could cause electrical loss (V_oc_ and FF), owing to the lattice mismatch and high defect densities at the p/i and i/n interfaces, while also causing optical loss for the bottom sub-cell. To restrain these losses, we employed buffer layers at the interfaces (to reduce electrical loss) and used a graded f-p band gap a-SiGe:H *i*-layer (to yield photon absorption). The buffer layers at the interfaces and the graded f-p band gap a-SiGe:H contributed to a 12.27% improvement in the J_sc_ of the tandem solar cells. Furthermore, we utilized a DL-ARC to gain photon absorption for the two sub-cells. Thanks to its excellent anti-reflective properties, this DL-ARC enabled a high J_sc_, and we thus confirmed that the incorporation of the DL-ARC at the front surface enhanced the efficiency of the tandem PV devices by up to 16.04%.

## Methods

We measured the optical constants and thickness of each of the layer using spectroscopic ellipsometry (VASE, Woollam, 240 nm < λ < 1700 nm) at room temperature. In order to simulate the single p/i/n-TFS device performance for the different a-SiGe:H active *i*-layers, we used the Advanced Semiconductor Analysis (ASA) computer program developed by Delft University of Technology^23^. We employed the Essential Macleod software package (Thin Film Center, Inc., Tucson, AZ, USA) to optimize the thickness of the MgF_2_ layer in the DL-ARC (MgF_2_/ITO) configuration. We performed the photo-generation rate with the help of the Automat FOR the Simulation of HETerostructures software (AFORS-HET 3.0.1)^29^.

We received the HIT-type bottom sub-cells from the Technology Engine of Science Co., LTD, Korea^30^. We deposited the top sub-cells with the n/i/p sequence in a cluster-type plasma-enhanced chemical vapor deposition system. We retained the thickness of the active *i*-layer of the top sub-cell at 600 nm. We deposited the 160 nm thick ITO film as a transparent front electrode by magnetron sputtering and using a suitable metal mask. We deposited the Ag/Al front finger-grids by thermal evaporation and the Al back-electrode by covering the entire rear side of the cell. To obtain the DL-ARC, we coated MgF_2_ layer on the ITO by thermal evaporation.

The current density-voltage characteristic curves were measured under AM 1.5 insolation, with 100 mW/cm^2^ light intensity at a temperature of 25 ^o^C, using a Keithley 2400 source meter. We investigated the pyramidal surface texture of the c-Si substrates and a cross-section of the top sub-cell on a rough texture similar to that of the bottom sub-cell by high resolution SEM (JSM-6300). We characterized the surface reflectance using a UV-Vis Spectrophotometer (Scinco S-3100). We measured the external quantum efficiency (EQE) of the tandem cell under a suitable optical and electrical bias at room temperature using a solar cell spectral response/QE/incident photo-to-current efficiency measurement system G1218a (PV Measurements, Inc., Boulder, CO, USA).
